# Immuno-histochemical correlation of fibrosis-related markers with the desmoplastic reaction of the mesentery in small intestine neuroendocrine neoplasms

**DOI:** 10.1007/s00432-022-04119-6

**Published:** 2022-07-07

**Authors:** Florian Bösch, Annelore Altendorf-Hofmann, Vanessa Koliogiannis, Harun Ilhan, Sven Jacob, Elise Pretzsch, Svenja Nölting, Jens Werner, Frederick Klauschen, Christoph J. Auernhammer, Martin K. Angele, Thomas Knösel

**Affiliations:** 1 Department of General, Visceral, and Transplant Surgery, University Hospital, LMU Klinikum, Ludwig-Maximilians-University Munich, Munich, Germany; 2 Department of General, Visceral Und Vascular Surgery, Friedrich-Schiller University, Jena, Germany; 3 Department of Radiology, University Hospital, LMU Munich, Ludwig-Maximilians-University Munich, Munich, Germany; 4 Department of Nuclear Medicine, University Hospital, LMU Klinikum, Ludwig-Maximilians-University Munich, Munich, Germany; 5 Department of Endocrinology, Diabetology and Clinical Nutrition, University Hospital Zurich (USZ) and University of Zurich (UZH), Zurich, Switzerland; 6 Department of Internal Medicine 4, University Hospital, LMU Klinikum, Ludwig-Maximilians-University Munich, Marchioninistr. 15, 81377 Munich, Germany; 7 Institute of Pathology, Ludwig-Maximilians-University, Munich, Germany; 8 Interdisciplinary Center of Neuroendocrine Tumors of the GastroEnteroPancreatic System (GEPNET-KUM), University Hospital, LMU Klinikum, Ludwig-Maximilians-University of Munich, Munich, Germany

**Keywords:** Neuroendocrine neoplasia, Small intestine, Fibrosis, Desmoplastic reaction, Mesentery

## Abstract

**Introduction:**

Small intestine neuroendocrine neoplasms (siNENs) will attain more importance due to their increasing incidence. Moreover, siNENs might lead to a desmoplastic reaction (DR) of the mesentery causing severe complications and deteriorating prognosis. The expression of fibrosis-related proteins appears to be the key mechanisms for the development of this desmoplastic reaction. Therefore, this study aimed to investigate the association of the desmoplastic mesentery with specific fibrosis-related protein expression levels.

**Materials and methods:**

By immunohistochemistry, the protein expression levels of four fibrosis-related markers (APLP2, BNIP3L, CD59, DKK3) were investigated in primary tumors of 128 siNENs. The expression levels were correlated with the presence of a desmoplastic reaction and clinico-pathological parameters.

**Results:**

In the primary tumor, APLP2, BNIP3L, CD59 and DKK3 were highly expressed in 29.7% (*n* = 38), 64.9% (*n* = 83), 92.2% (*n* = 118) and 80.5% (*n* = 103), respectively. There was no significant correlation of a single marker or the complete marker panel to the manifestation of a desmoplastic mesentery. The desmoplastic mesentery was significantly associated with clinical symptoms, such as flushing and diarrhea. However, neither the fibrosis-related marker panel nor single marker expressions were associated with clinical symptoms.

**Discussion:**

The expression rates of four fibrosis-related markers in the primary tumor display a distinct pattern. However, the expression patterns are not associated with desmoplastic altered mesenteric lymph node metastases and the expression patterns did not correlate with prognosis. These findings suggest alternative mechanisms being responsible for the desmoplastic reaction.

## Introduction

Small intestine neuroendocrine neoplasms (siNENs) have an increasing incidence (Lawrence et al. 2011). Thus, this entity will attain increasing importance in daily clinical routine. Previously it has been demonstrated that siNEN might lead to a desmoplastic reaction (DR) of the mesentery (Blazevic et al. 2018; Bosch et al. 2018; Koumarianou et al. 2018; Laskaratos et al. 2021, 2020a). The desmoplastic mesentery shows fibrotic alterations, often leads to symptoms and its appearance in cross-sectional imaging modalities is pathognomonic for siNEN (Rodriguez Laval et al. 2018). Since the DR is closely related to the main visceral vessels, it might be associated with ischemia of the small intestine and/or cause intestinal obstruction (Makridis et al. 1997). Moreover, siNENs causing a DR of the mesentery tend to be biologically more aggressive and are associated with a deteriorated oncological prognosis (Bosch et al. 2018; Laskaratos et al. 2020a). Although DR is present in about one-third of patients with siNEN, profound knowledge of the underlying mechanisms is still lacking. Determination of markers associated with the development of DR may be of interest for the assessment of prognosis and for the detection of novel therapeutic targets.

It has been assumed that peptides secreted by siNENs induce DR or that an infiltration of blood vessels causes the fibrotic changes (Makridis et al. 1997; Ohrvall et al. 2000). Furthermore, the microenvironment of neuroendocrine tumors including various growth factors and mediators, such as connective tissue growth factor, fibroblast growth factor or TGF-beta1, has also been discussed to stimulate DR (Koumarianou et al. 2018; Cunningham et al. 2010; Kidd et al. 2007; Rosa et al. 2001). Transcriptome profiling identified the integrin signaling pathway to be involved in DR (Laskaratos et al. 2021). The mRNA-based multi-analyte NETest analyzes the expression rates of 51 gene transcripts in blood samples and has a superior sensitivity and specificity in diagnosing gastro-entero-pancreatic NENs (GEP-NENs) (Malczewska et al. 2020, 2021). Recently, in blood samples from 20 siNEN patients, a subset of five circulating gene transcripts from the NETest were significantly associated with mesenterial fibrosis (Laskaratos et al. 2020a). These five genes were called the ROC curve analysis and demonstrated the most significant prediction of mesenterial fibrosis by APLP2 (amyloid precursor-like protein 2), BNIP3L (BCL2-Interacting Protein 3 Like) and CD59 (CD 59 glycoprotein, syn. MAC inhibitory protein) (6). Moreover, DKK-3 (Dickkopf-related protein 3) is known to play an important role in cardiac fibrosis (Zeng et al. 2021) and renal fibrosis (Lipphardt et al. 2019). To our knowledge, however, the role of DKK-3 in fibrosis in NENs has not been investigated so far.

The present study aimed to evaluate the association of the expression patterns of distinct markers of fibrosis, including markers of the fibrosome, within the primary tumors of a comprehensive and well-characterized cohort of siNEN patients. Moreover, since DR is associated with a deteriorated outcome, the prognostic relevance of the fibrosis markers needs to be analyzed. Thus, a correlation of the expression levels of fibrosis markers in the primary tumor and the fibrotic mesentery can be investigated and a biomarker might be determined identifying patients at risk for DR.

## Materials and methods

Patients undergoing surgery for a siNEN at the Department of General, Visceral, and Transplant Surgery at the Ludwig-Maximilians-University Munich, Munich, Germany, were entered into a prospectively led database. As described previously, patients were screened for the presence or absence of a desmoplastic altered mesentery and divided into two groups (Bosch et al. 2018). Thus, a preoperative cross-sectional imaging modality was required. Furthermore, to confirm radiological DR, the histopathological sections were evaluated for fibrotic changes within the mesentery (Bosch et al. 2018). Based on this evaluation, the patients were classified into the two groups. The scans were screened by one independent and blinded radiologist (V.K.) and one blinded and independent nuclear medicine physician (H.I.). Moreover, for immuno-histochemical analyses, a tissue microarray (TMA) of the primaries was used and thus a specimen of the primary tumor was required. In brief, out of each siNEN primary tumor, two punch biopsies with a diameter of 0.6 mm were taken from selected areas and mounted on a conventional microscope slide for staining and analysis (Bosch et al. 2020, 2019). Hence, the present study included 128 patients.

Immunohistochemistry was performed with commercially available antibodies against APLP2 (amyloid precursor-like protein 2; HPA039319, Atlas Antibodies AB, Bromma, Sweden), BNIP3L (BCL2-Interacting Protein 3 Like; HPA015652, Atlas Antibodies AB, Bromma, Sweden), CD59 (HPA026494, Atlas Antibodies AB, Bromma, Sweden) and DKK3 (Dickkopf 3; 10,365-1-AP, Proteintech Europe, Manchester, United Kingdom) (see Table 1). All antibodies were raised in rabbit and the slides were incubated with the primary antibody for 60 min at room temperature. The used dilutions for the primary antibodies were as follows: APLP2 1:100, BNIP3L 1:150, CD59 1:100, DKK3 1:100. Pretreatment consisted of heat and the use of different antigen enhancers; slides for APLP2 detection were pretreated with the Target Unmasking Fluid (PanPath), for BNIP3L detection with the ProTaqs V Antigen-Enhancer (Quartett), for CD59 detection with the Epitope Retrieval Solution pH8 Novocastra (Leica Biosystems) and for DKK3 detection with the ProTaqs I Antigen-Enhancer (Quartett). For APLP2 and BNIP3L, the chromogen AEC + (Agilent) and for CD59 and DKK3, the chromogen DAB + (Agilent) was used for detection in combination with the ImmPRESS Reagent Kit (Vector Laboratories). The TMA blocks were cut at 5 µm sections and immuno-histochemical staining was done as described previously on a Ventana Benchmark XT autostainer system (Bosch et al. 2020, 2019; Kampmann et al. 2015). The tumor spots were assessed and confirmed with a hematoxylin and eosin staining. Furthermore, by mounting control spots, unspecific staining could be ruled out.

**Table 1:** Antibodies and dilution used

Antibody	Dilution	Pretreatment	Chromogen
APLP2	1:100	Target unmasking fluid	AEC +
BNIP3L	1:150	ProTaqs V antigen-enhancer	AEC +
CD59	1:100	Epitope retrieval solution pH8 Novocastra	DAB +
DKK3	1:100	ProTaqs I antigen-enhancer	DAB +

**Fig. 1:**
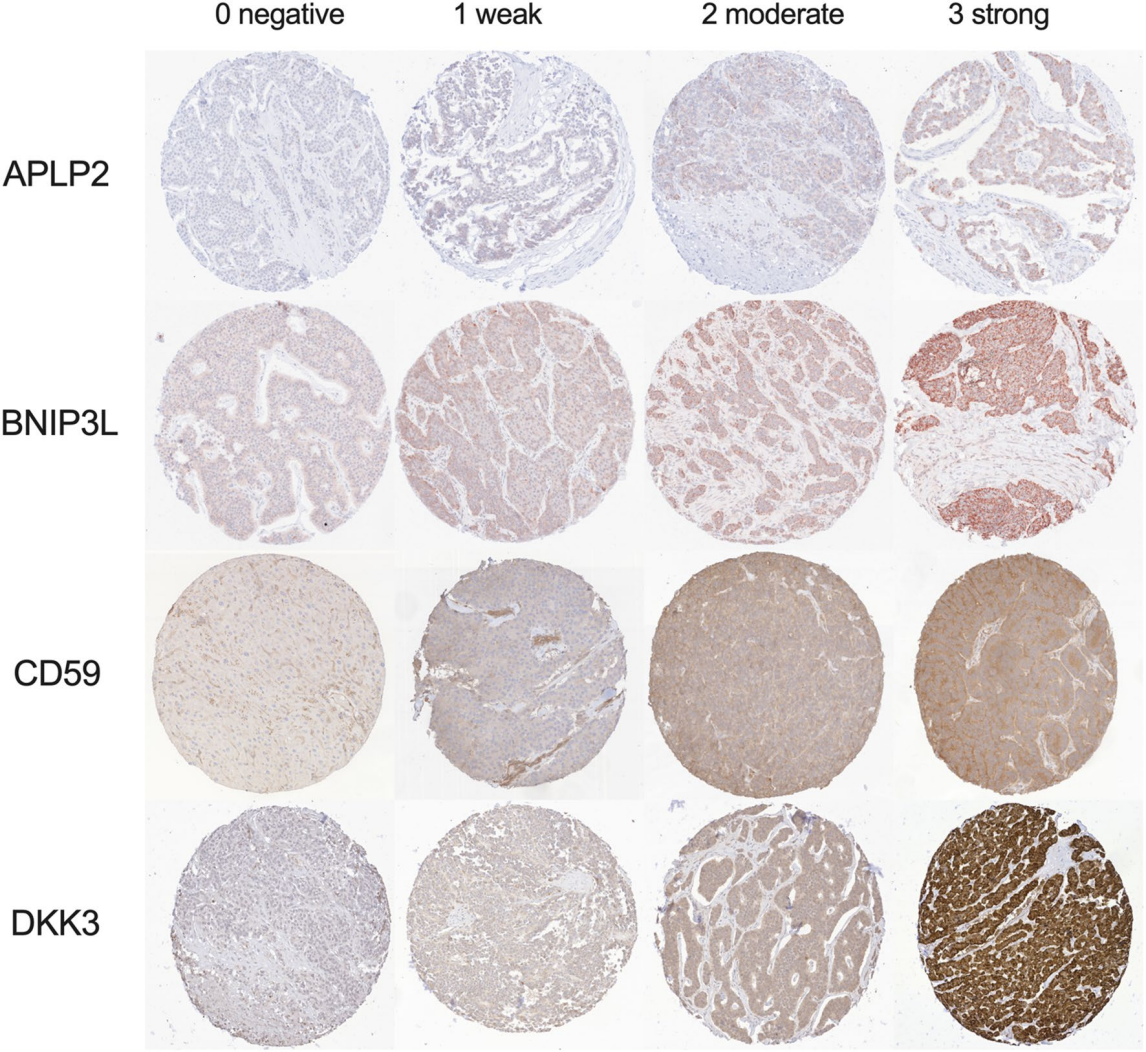
Presentation of the different expression rates of the tested markers

The immuno-histochemical staining was assessed by two blinded and independent observers (F.B., T.K.). Every punch was categorized semi-quantitatively into 0: negative; 1: weakly positive; 2: moderately positive; 3: strongly positive (see Fig. 1). If necessary, the slides were re-evaluated under a multi-headed microscope and consensus reached. To attain a more robust conclusion considering the sample size, expression rates were grouped into no/low (negative and weakly positive) and high (moderately and strongly positive).

All statistical analyses were performed using SPSS 26.0 (IBM, Chicago, IL, USA) software. Categorical variables were tested for independence using the Fisher’s exact test. Survival was calculated from the date of surgery. End points for observed and disease-free survival were death of any cause and diagnosis of first recurrence, respectively. Significant and independent predictors of overall survival and disease-free survival were identified by Cox proportional hazard analysis. The procedure was set to a threshold of 0.05. Statistical significance was defined as a *p *value < 0.05 for all analyses.

## Results

In the present analysis, the primary tumors of 128 siNEN patients were analyzed. The clinicopathological characteristics are displayed in (Table 2). The median age of the study cohort was 62.5 years (Range: 30–81 years), 77 males and 51 females were included. A desmoplastic altered mesentery was present in 55 patients (43%). All tumors were G1 / G2, a majority of 67.2% (*n* = 86) of patients had a G1 tumor. DR was significantly associated with distant metastases and with a perineural tissue infiltration (*p* < 0.05). However, there was neither a significant correlation of DR with the tumor size nor its grading or Ki67 index (*p* > 0.05).

**Table 2:** Univariate analysis of clinicopathological characteristics of the analyzed cohort using the Fisher’s exact test

Variable	Desmoplastic reaction *n* = 55 (43%)	No desmoplastic reaction *n* = 73 (57%)	*p *value
Gender			** < 0.05**
Female	39 (70.9%)	38 (52.1%)	
Male	16 (29.1%)	35 (47.9%)	
Grading			> 0.05
G1	39 (70.9%)	47 (64.4%)	
G2	16 (29.1%)	26 (35.6%)	
Tumor stage			> 0.05
T 1/2	9 (16.4%)	23 (31.9%)	
T 3/4	46 (83.6%)	49 (68.1%)	
Distant metastasis			** < 0.05**
Yes	45 (81.8%)	30 (41.1%)	
No	10 (18.2%)	43 (58.9%)	
Lymphatic vessel infiltration			> 0.05
Yes	35 (63.6%)	34 (46.6%)	
No	20 (36.4%)	39 (53.4%)	
Perineural tissue infiltration			** < 0.05**
Yes	32 (58.2%)	20 (27.4%)	
No	23 (41.8%)	53 (72.6%)	
Angio-invasion			> 0.05
Yes	10 (18.2%)	13 (17.8%)	
No	45 (81.8%)	60 (82.2%)	
Flushing			** < 0.05**
Yes	21 (38.2%)	14 (19.2%)	
No	34 (61.8%)	59 (80.8%)	
Diarrhea			** < 0.05**
Yes	23 (41.8%)	16 (21.9%)	
No	32 (58.2%)	57 (78.1%)	

A *p *value < 0.05 was considered as statistically significant (in bold)

The staining of all four investigated markers was conclusive for every specimen. The expression rates of the four markers were heterogeneously distributed. siNENs were highly expressing CD59, DKK3 and BNIP3L in 92.2% (*n* = 118), 80.5% (*n* = 103) and 64.9% (*n* = 83), while only 38 tumors (29.7%) highly expressed APLP2. However, there was no significant correlation of the expression rates of either marker with the presence of a DR (*p* > 0.05). In a further analysis, the predictive value of the combination of the four markers was tested. This analysis revealed that only 20 cases (15.6%) had high expression rates of all four markers. Thus, there was no significant correlation of the complete marker panel to DR accordingly (*p* > 0.05). High APLP2 was seen significantly more often in tumors with infiltration of the perineural tissue (*p* < 0.05) (see Table 3).

**Table 3:** Cross-table showing the correlation of APLP2, BNIP3L, CD59 and DKK3 to the presence of desmoplastic reaction analyzed with the Fisher’s exact test

Marker	Desmoplastic reaction	No desmoplastic reaction	*p *value
APLP2			> 0.05
Low	36 (40%)	54 (60%)	
High	19 (50%)	19 (50%)	
BNIP3L			> 0.05
Low	19 (42%)	26 (58%)	
High	36 (43%)	47 (57%)	
CD59			> 0.05
Low	7 (70%)	3(30%)	
High	48 (41%)	70 (59%)	
DKK3			> 0.05
Low	8 (32%)	17 (68%)	
High	47 (46%)	56 (54%)	

A *p *value < 0.05 was considered as statistically significant

The univariate survival analysis demonstrated that siNENs leading to a desmoplastic mesentery were associated with significantly deteriorated survival rates (*p* < 0.05). Nonetheless, DR was not an independent risk factor for overall survival in the multivariate analysis (*p* > 0.05). Patients with a DR developed recurrent disease significantly earlier (median 18 months) than patients without a DR (median 42 months) (*p* < 0.001). DR was an independent risk factor for recurrence-free survival. Additionally, the presence of distant metastases at diagnosis was an independent risk factor for a significantly shorter recurrence-free survival (see Table 4).

**Table 4:** Univariate and multivariate Cox regression analysis for disease-free survival

Variable	Univariate analysis		Multivariate analysis	
	Hazard ratio	*p *value	Hazard ratio	*p *value
Grading	1.369 (0.830–2.259)	0.218		
Tumor stage	3.605 (1.711–7.596)	**0.001**	1.389 (0.610–3.163)	0.434
Lymph node metastasis	0.805 (0.397–1.631)	0.547		
Distant metastasis	7.884 (3.957–15.707)	**0.000**	4.265 (1.891–9.619)	**0.000**
Lymphatic vessel infiltration	1.994 (1.207–3.292)	**0.007**	1.391 (0.722–2.682)	0.324
Angio-invasion	2.798 (1.613–4.854)	**0.000**	1.626 (0.839–3.150)	0.150
Perineural tissue infiltration	2.197 (1.345–3.589)	**0.002**	1.012 (0.524–1.952)	0.972
Flushing	2.453 (1.490–4.039)	**0.000**	1.076 (0.611–1.896)	0.799
Diarrhea	2.106 (1.282–3.459)	**0.003**	1.437 (0.809–2.552)	0.216
APLP2	1.363 (0.814–2.284)	0.239		
BNIP3L	0.975 (0.590–1.611)	0.922		
CD59	0.941 (0.342–2.587)	0.906		
DKK	1.517 (0.750–3.066)	0.246		
Desmoplastic reaction	2.818 (1.710–4.642)	**0.000**	1.763 (1.025–3.032)	**0.040**

For effect size, the hazard ratio with its 95% confidence intervals and *p *values were reported. A *p *value < 0.05 was considered as statistically significant (in bold)

Data of preoperatively measured biomarkers, such as Chromogranin A (CgA), serotonin and 5-hydroxyindoleacetic acid (5-HIAA), were analyzed. The values of CgA were available for 100 patients (78.1%), of serotonin for 30 patients (23.4%) and of 5-HIAA for 84 patients (65.6%). The information about clinical symptoms, such as flushing and diarrhea, was conclusive for the entire cohort. Therefore, only the clinical symptoms and CgA as well as 5-HIAA were further investigated. None of the four fibrosis-related markers showed a significant correlation to clinical symptoms (*p* > 0.05). However, DR was significantly associated with flushing and diarrhea. Flushing was present in 60% (*n* = 21) of patients with DR and 59% of patients with DR (*n* = 23) suffered of diarrhea. CgA blood levels did not correlate significantly with a fibrosis-related marker or the presence of DR. On the contrary, 5-HIAA blood levels were significantly increased in patients with either a DR or a siNEN highly expressing APLP2 (*p* < 0.05).

## Discussion

Due to the steadily increasing incidence of GEP-NENs, these tumors demand more awareness. The algorithms of treating GEP-NENs are mainly based on (small) retrospective studies and the most important prognostic factors remain tumor size and grading (Partelli et al. 2017; Perren et al. 2017). Moreover, there are no reliable diagnostic or predictive biomarkers with a high sensitivity and specificity. The most common peptide used as a biomarker is CgA, which in turn has a low accuracy and is influenced by a variety of clinical conditions and medication (Giacinto et al. 2018; Pregun et al. 2011). In this respect, the NETest, analyzing the expression levels of 51 genes in blood samples, demonstrated to be a reliable diagnostic tool with a diagnostic accuracy above 90% (Malczewska et al. 2020, 2021). Moreover, studies suggest that the NETest facilitates to assess efficacy and completeness of surgical resection (Laskaratos et al. 2020b; Partelli et al. 2020).

Recently, Laskaratos et al. defined in blood samples of 20 siNEN patients a subset of five gene transcripts derived from the 51 mRNA multi-analyte NETest and called it fibrosome. The authors described a significant association of the transcript expression levels of the fibrosome markers APLP2, BNIP3L, CD59 and CTGF and the presence of mesenteric fibrosis (Laskaratos et al. 2020a). In the present study, the three markers from the fibrosome with the highest reported predictive values (APLP2, BNIP3L, CD59) (Laskaratos et al. 2020a) and an additional well-known fibrosis marker (DKK3) in renal (Lipphardt et al. 2019) and cardiac disease (Zeng et al. 2021; Zhai et al. 2018) were investigated on protein expression level in primary tumor samples.

We observed no significant correlation of these markers to DR. The lack of significance might be due to the fact that gene expression levels as reported by Laskaratos et al. (Laskaratos et al. 2020a) differ from protein expression levels measured by immunohistochemistry like in the present study (Pascal et al. 2008). Nonetheless, analyzing protein expression levels closes the gap between transcribed genes and produced proteins. Thus, immunohistochemistry provides a direct view on the activity and productivity of a tumor. Moreover, Laskaratos et al. analyzed only 20 serum samples compared to this study with an immuno-histochemical analysis of 128 primary tumors, which supports the present results. Nonetheless, the expression levels of the investigated proteins might be heterogeneous between tumor, tumor progression zone and tumor microenvironment and therefore not be detected in TMA analysis of tumor samples. Moreover, the elevated serum expression levels of the fibrosome genes in the study by Laskaratos et al. might be influenced by the desmoplastic mesentery itself. In this respect, an analysis of gene expression levels of the primary tumor is lacking including RNA as well microRNA expression levels. Thus, further studies investigating fibrosis markers and their association to DR are urgently needed.

On the other hand, the present study analyzed only three out of five fibrosome markers. But these three markers had the highest predictive power with an area under the curve (AUC) in the receiver operating characteristic analysis of 0.962–0.969 compared to the other two fibrosome genes with an AUC of only 0.377 and 0.785 (Laskaratos et al. 2020a). Therefore, it seems appropriate to combine these three markers with an AUC of almost 1. In addition, we investigated the expression of DKK3 in siNENs, as DKK3 has been reported to be involved in the development of renal and cardiac fibrosis (Zeng et al. 2021; Lipphardt et al. 2019), and its role in tumorigenesis has been investigated in various cancers (Kim et al. 2013; Lee et al. 2020). While DKK3 expression is downregulated in the majority of cancers, also upregulation of DKK3 has been observed in some types of cancer (Lee et al. 2020). Also, the role of DKK3 in tumorigenesis varies in different cancers. On the one hand, DKK3 has been shown to act as a tumor suppressor in many cancers and DKK3 overexpression has been investigated as a therapeutic target including a current phase I trial of adenovirus-vector-mediated DKK3 overexpression in liver metastases of hepatocellular carcinoma. On the other hand, DKK3 has also been reported to stimulate tumor-promoting cancer-associated fibroblasts (Ferrari et al. 2019) and suppression of DKK3 has been shown to inhibit tumor growth in pancreatic ductal adenocarcinoma (Zhou et al. 2018). In human neuroendocrine BON1 cells, DKK3 expression was shown to be epigenetically downregulated and overexpression of DKK3 inhibited colony formation of BON1 cells (Kim et al. 2013). In contrast, in the current study using TMA analysis, we observed high expression levels of DKK3 in 80% of siNENs. Thus, the potential role of DKK3 in the tumorigenesis of NENs and as a potential therapeutic target should be further investigated in future studies.

The present study underlined that tumors leading to DR are biologically more aggressive. Patients with a DR had significantly shorter recurrence-free survival rates and these tumors showed significantly more often a perineurial tissue infiltration. Nonetheless, DR was not associated to the well-established risk factors grading and tumor size, which indicates that tumors leading to a desmoplastic mesentery bear an independent enhanced malignant potential. In addition, DR was highly significantly associated with the presence of distant metastases, which in turn were also an independent prognostic factor. Thus, it is pivotal to identify patients at risk who will develop a DR. Since there was no correlation between the analyzed marker panel and the presence of DR, the present study does not support the idea that the analyzed markers induce the desmoplastic reaction of the mesentery. Moreover, siNENs expressed in approximately 65–92% three of the four markers disregarding DR, which in turn was present in 43% of siNENs. These findings suggest that the marker panel is abundantly expressed in siNENs and, thus, might not be a good candidate in predicting DR. However, due to the abundance, it seems that specific markers might be helpful diagnosing siNENs.

SiNEN patients with a desmoplastic mesentery are significantly more prone to flushing and diarrhea. Thus, a possible correlation of the marker panel with clinical symptoms and biomarkers, such as CgA, serotonin and 5-HIAA, was investigated. Due to incomplete data sets, only CgA and 5-HIAA could be analyzed sufficiently. Nonetheless, the protein expression levels of the fibrosis-related markers were not associated to CgA and 5-HIAA blood levels. This lack of significance might be due to the fact that the present study analyzed the fibrosis-related markers in the primary tumor. Furthermore, only less than a third of patients expressed APLP2, which is contradictory to Arvidsson et al. who found 81% (*n* = 25/31) of siNENs expressing APLP2 (Arvidsson et al. 2008). Intriguingly, 5-HIAA levels were significantly associated with the presence of DR and APLP2 expressing tumors. Thus, further studies are necessary investigating distinct panels combining various markers and diagnostic approaches. Furthermore, it is required to analyze the primary tumor on RNA and microRNA levels to characterize the effect of fibrosis markers on the development of the desmoplastic reaction.

Although this is the largest study of a well-described siNEN collective so far, there are limitations as well. First, this is a retrospective cohort study of a surgical collective introducing a bias. Since siNENs are still a rare disease and diagnostic as well as predictive tools need to be discovered, retrospective studies are inevitable. The present study presents comprehensive data of more than 120 siNEN patients representing the largest collective in this field. Second, the present analysis investigated histological sections on a TMA which represents only a small part of the tumor. Thus, to minimize a bias, two punch biopsies of each primary tumor were analyzed and using a TMA is well established (Kidd et al. 2007; Bosch et al. 2020, 2019; Kampmann et al. 2015). However, the expression levels of the investigated proteins might be heterogeneous between tumor, tumor progression zone and tumor microenvironment and therefore not be detected in TMA analysis of tumor samples.

In conclusion, this is the first study elucidating the association of four well-known fibrosis-related markers to the presence of a desmoplastic mesentery. However, there was no significant correlation of the marker panel to the prognostic relevant DR. Thus, the induction of DR remains ill-defined and further research is urgently necessary.

## Abbreviations

5-HIAA: 5-Hydroxyindoleacetic acid
95% CI: 95% Confidence interval
APLP2: Amyloid precursor-like protein 2
AUC: Area under the curve
BNIP3L: BCL2-Interacting Protein 3 Like
DKK3: Dickkopf 3
CgA: Chromogranin A
GEP-NEN: Gastroenteropancreatic neuroendocrine neoplasm
NEN: Neuroendocrine neoplasm
siNEN: Small intestine neuroendocrine neoplasm
TMA: Tissue microarray
